# Structure and inhibitor specificity of the PCTAIRE-family kinase CDK16

**DOI:** 10.1042/BCJ20160941

**Published:** 2017-02-20

**Authors:** Sarah E. Dixon-Clarke, Saifeldin N. Shehata, Tobias Krojer, Timothy D. Sharpe, Frank vonDelft, Kei Sakamoto, Alex N. Bullock

**Affiliations:** 1 Structural Genomics Consortium, University of Oxford, Old Road Campus, Roosevelt Drive, Oxford OX3 7DQ, U.K.; 2 Nestlé Institute of Health Sciences SA, EPFL Innovation Park, bâtiment G, 1015 Lausanne, Switzerland; 3 School of Life Sciences, Ecole Polytechnique Fédérale de Lausanne (EPFL), 1015 Lausanne, Switzerland; 4 Diamond Light Source Ltd, Harwell Science and Innovation Campus, Didcot OX11 0QX, U.K.; 5 Department of Biochemistry, University of Johannesburg, Auckland Park 2006, South Africa

**Keywords:** CCNY, cyclin, kinase, PCTAIRE1, PCTK1, phosphorylation

## Abstract

CDK16 (also known as PCTAIRE1 or PCTK1) is an atypical member of the cyclin-dependent kinase (CDK) family that has emerged as a key regulator of neurite outgrowth, vesicle trafficking and cancer cell proliferation. CDK16 is activated through binding to cyclin Y via a phosphorylation-dependent 14-3-3 interaction and has a unique consensus substrate phosphorylation motif compared with conventional CDKs. To elucidate the structure and inhibitor-binding properties of this atypical CDK, we screened the CDK16 kinase domain against different inhibitor libraries and determined the co-structures of identified hits. We discovered that the ATP-binding pocket of CDK16 can accommodate both type I and type II kinase inhibitors. The most potent CDK16 inhibitors revealed by cell-free and cell-based assays were the multitargeted cancer drugs dabrafenib and rebastinib. An inactive DFG-out binding conformation was confirmed by the first crystal structures of CDK16 in separate complexes with the inhibitors indirubin E804 and rebastinib, respectively. The structures revealed considerable conformational plasticity, suggesting that the isolated CDK16 kinase domain was relatively unstable in the absence of a cyclin partner. The unusual structural features and chemical scaffolds identified here hold promise for the development of more selective CDK16 inhibitors and provide opportunity to better characterise the role of CDK16 and its related CDK family members in various physiological and pathological contexts.

## Introduction

CDK16 is a newly recognised member of the CDK protein kinase family [1,2] that is activated upon binding to the membrane-associated protein cyclin Y (CCNY) or its homologue cyclin Y-like 1 (CCNYL1) [3–5]. These interactions are facilitated by a ‘PCTAIRE’ sequence motif in the kinase αC helix that is conserved in CDK17 and CDK18, but diverged from the classical ‘PSTAIRE’ motif found in CDK2 [6]. CDK16 and cyclin Y contain central kinase and cyclin box domains, respectively, both flanked by N- and C-terminal extensions that form sites of regulatory phosphorylation [5,7]. Phosphorylations at Ser100 and Ser326 in cyclin Y promote an additional interaction with 14-3-3 protein that also appears necessary for its binding to and activation of CDK16 [5,8].

CDK16 is ubiquitously expressed, but is particularly high in terminally differentiated cells and in the brain, where it is suggested to control neurite outgrowth [7,9]. In addition, CDK16 is highly expressed in the testis where it plays a unique role in the terminal steps of spermatogenesis [3,4]. Genetic knockdown in *Caenorhabditis elegans* has suggested parallel roles for a CDK16-18 orthologue (PCT-1) and CDK5 in the inhibition of retrograde axonal trafficking [10]. CDK16 has also been implicated in other diverse processes, including vesicle trafficking [11,12], glucose homeostasis [13,14] and muscle differentiation [15].

In addition to these important biological functions, CDK16 has been implicated in the growth of several cancers [16] and its expression has been found to be significantly elevated in tissues derived from prostate and breast cancers [17]. In agreement, siRNA-mediated knockdown of CDK16 has been shown to inhibit the proliferation of medulloblastoma, prostate, breast, melanoma and cervical cancer cell lines [16,18,19]. Furthermore, CDK16 knockdown reduced tumour volume in mouse xenograft models of colorectal cancer [20]. Interestingly, CDK16 knockdown did not affect proliferation in non-transformed cells [16]. Taken together, these data identify CDK16 as a potential target for the development of novel anti-cancer drugs. However, the mechanism by which CDK16 is involved in cancer cell growth is unknown and selective small-molecule inhibitors for CDK16 have not been identified.

Here, we show that the kinase domain of CDK16 can bind to a diverse set of chemical inhibitor scaffolds, but has a broad preference for known CDK inhibitors, consistent with its sequence homology. Of note for future chemistry efforts, both type I and type II kinase inhibitors are among the most potent CDK16 inhibitors, as exemplified by the clinically tested compounds dabrafenib and rebastinib, respectively. We further confirm that these compounds can bind to full-length (FL) CDK16 in intact cells. In addition, we report the first crystal structures of CDK16 in separate complexes with the inhibitors indirubin E804 and rebastinib, respectively. The structures reveal considerable conformational plasticity. In particular, the partial unfolding of the αC helix in the indirubin E804 co-structure suggests that the isolated CDK16 kinase domain may be relatively unstable in the absence of a cyclin partner. Potentially, this unusual αC structure could be exploited in future to develop more selective CDK16 inhibitors.

## Materials and methods

### Materials

PCTAIRE-tide (PKSPKARKKL) peptide substrate for CDK16 kinase assays was synthesised by GL Biochem. [^γ-32^P]ATP was obtained from PerkinElmer. Cell culture reagents were obtained from Life Technologies. Preclinical kinase inhibitors were obtained from Calbiochem. Clinical kinase inhibitors were obtained from the FIMM drug collection. Published kinase inhibitor set (PKIS) compounds were a gift from William Zuercher (GlaxoSmithKline). P81 paper was obtained from Whatman. Unless otherwise indicated, all other reagents were obtained from Sigma.

### Antibodies

Anti-CDK16 antibody (HPA001366) was obtained from Sigma. Anti-glyceraldehyde-3-phosphate dehydrogenase (GAPDH) (G9) antibody (sc-365062) was obtained from Santa Cruz Biotechnology. Anti-haemagglutinin (HA) antibody (HA.11) was from Covance Research Products and anti-FLAG antibody (F7425) was from Sigma. Site-specific rabbit polyclonal antibodies against phospho-cyclin Y (pSer12, pSer100, pSer326 and pSer336) were described recently [5]. Horseradish peroxidase-conjugated secondary antibodies used in Figures 3 and 4 were from Jackson ImmunoResearch. Anti-rabbit IgG (Sigma, #A6667) and anti-mouse IgG (Dako) were used in experiments shown in Figure 5.

### Plasmids

All plasmid constructs were generated using standard molecular biology techniques. Cloning and mutagenesis of the coding regions of CDK16 isoform 1 (NM_006201.4) and cyclin Y (NM_145012.4) used in Figures 2–4 have been described recently [5]. Briefly, FL CDK16 was cloned into the mammalian expression vector pCMVFLAG-2, whereas FL cyclin Y was cloned into pCMVHA-1. The kinase-inactive D304A mutant of CDK16 and phospho-deficient S336A mutant of cyclin Y were prepared using the QuikChange method (Agilent) and KOD Hot Start DNA Polymerase (Novagen). For structural and inhibitor screening work, human CDK16^163–478^ with the phosphomimetic S319D mutation was cloned into the bacterial expression vector pNIC-CH, which encodes for a non-cleavable C-terminal hexahistidine tag.

### Protein expression and purification

Protein expression used the *Escherichia coli* strain BL21 (DE3) additionally transformed with the plasmid pRARE2. Cultures were grown at 37°C to the mid-log phase in Luria–Bertani broth media and protein expression was induced overnight at 18°C with 0.5 mM isopropyl β-d-1-thiogalactopyranoside. Cells were harvested by centrifugation and lysed by ultrasonication in a buffer containing 50 mM HEPES (pH 7.5), 500 mM NaCl, 5% glycerol, 5 mM imidazole, 1 mM phenylmethylsulphonyl fluoride (PMSF), 0.5 mM tris(2-carboxyethyl)phosphine (TCEP). The cell lysate was then clarified by centrifugation. CDK16 protein was purified initially using nickel sepharose chromatography (GE Healthcare) and eluted stepwise with imidazole. Further purification was performed by size-exclusion chromatography using a HiLoad Superdex S200 16/60 column (GE Healthcare) buffered in 50 mM HEPES (pH 7.5), 300 mM NaCl and 0.5 mM TCEP. For crystallisation with indirubin E804, a final clean-up step was also performed by anion exchange chromatography using a monoQ column buffered in 50 mM Tris (pH 9.0) with a gradient of 0–1.0 M NaCl. The correct intact mass of the protein was confirmed by mass spectrometry.

### Differential scanning fluorimetry

Thermal melting experiments were performed using a Real-Time PCR machine Mx3005p (Stratagene) as described previously [21]. Briefly, 2 μM CDK16 was screened against a collection of kinase inhibitors in a 96-well plate format. Compounds were added to a final concentration of 12.5 μM in an assay buffer containing 10 mM HEPES (pH 7.4), 150 mM NaCl and SYPRO Orange (1:1000 dilution, Sigma). Fluorescence was monitored as samples were heated from 25 to 96°C. Data were analysed with the MxPro software. Thermal shift values induced by inhibitor binding were calculated relative to control wells containing protein and 2.5% dimethyl sulfoxide (DMSO).

### *In vitro* CDK16 kinase assay

*In vitro* CDK16 kinase assay was performed as described recently [5]. *E. coli*-purified human CDK16 (125 ng) was incubated with varying concentrations of compound for 5 min followed by the addition of COS1-purified FLAG-human cyclin Y (125 ng) bound to endogenous 14-3-3 and assayed for phosphotransferase activity at 30°C for 30 min in a final assay volume of 50 µl containing 50 mM HEPES (pH 7.5), 0.1 mM EGTA, 10 mM magnesium acetate, 0.1 mM [^γ-32^P]ATP (300 c.p.m./pmol), 0.03% Brij 35, 1 mM dithiothreitol (DTT) and 50 µM of the CDK16 substrate PCTAIRE-tide (PKSPKARKKL) [22]. Reactions were terminated by spotting onto P81 paper and immersion in 75 mM H_3_PO_4_. After washing with H_3_PO_4_, P81 papers were air-dried and incorporation of ^γ-32^P was determined by Cherenkov counting. IC_50_ values were determined using three independent experiments.

### Cellular CDK16 kinase assay and cyclin Y binding

COS1 cells were maintained in high-glucose Dulbecco's modified Eagle's medium supplemented with 10% (v/v) foetal bovine serum (FBS) under 5% CO_2_. Cells were transfected with DNA constructs [wild-type (WT) or mutant of human FLAG-CDK16 and HA-cyclin Y] using polyethyleneimine and 24 h later treated with varying concentrations of compound for 1 h. Cells were then washed with ice-cold phosphate-buffered saline (PBS) and scraped into lysis buffer [50 mM Tris–HCl (pH 7.5), 1 mM EDTA, 1 mM EGTA, 270 mM sucrose, 1% (w/v) Triton X-100, 50 mM NaF, 5 mM Na_4_P_2_O_7_, 1 mM Na_3_VO_4_, 1 mM DTT, 1 mM benzamidine and 0.5 mM PMSF]. Lysates were centrifuged at 17 000 ***g*** for 10 min at 4°C and stored at −80°C. Protein concentration was determined using Bradford reagent and BSA standard. Lysates were immunoblotted with the CDK16 kinase activity-dependent (Ser336) or -independent (Ser12, Ser100 and Ser326) site-specific phospho-cyclin Y antibodies as the surrogate marker for specific and non-specific cellular CDK16 activity [5]. The compound-treated lysates were also immunoprecipitated using FLAG-agarose and immunoblotted using HA (cyclin Y) and 14-3-3 antibodies to assess the effect of the compounds on CDK16–cyclin Y binding.

### Cellular thermal shift assay

Cellular thermal shift assay (CETSA) experiments were performed as described previously [23,24]. IGR-37 melanoma cells were seeded into a T75cm^2^ flask at a density of 5 × 10^6^ cells/ml. After 24 h, the cells were treated for 30 min with cell media [RPMI supplemented with 10% (v/v) FBS and 1% penicillin/streptomycin] containing either 20 µM rebastinib or vehicle (0.08% DMSO). After treatment, cells were collected by centrifugation and washed in 1× PBS (5.5 ml). The cells were resuspended in 1 ml of PBS and the cell suspension was aliquoted at 90 μl into PCR tubes. Following further centrifugation, all 1× PBS was removed before the cells were heated for 3 min at 37, 41, 45, 49, 53, 57, 61, 65, 69 or 73°C. Subsequently, cells were lysed in 120 µl of lysis buffer [50 mM Tris–HCl (pH 7.5), 100 mM NaCl, 0.8% NP-40, 5% glycerol, 1.5 mM MgCl_2_, 25 mM NaF, 1 mM Na_3_VO_4_, 1 mM PMSF, 1 mM DTT, 10 µg/ml TLCK, 1 µg/ml leupeptin, 1 µg/ml aprotinin and 10 µg/ml soybean trypsin inhibitor (Sigma, pH 7.5)] by three consecutive freeze–thaw cycles using liquid nitrogen. The soluble fraction was separated from precipitated materials by centrifugation at 16 200 g and 4°C for 20 min. The supernatant, containing the soluble proteins, was transferred to a fresh tube and analysed by immunoblotting (IB).

### Crystallisation

For the indirubin E804 complex, CDK16 protein was buffered in 25 mM HEPES (pH 7.5), 250 mM NaCl, 5% glycerol and 10 mM DTT. Protein was concentrated to 15 mg/ml in the presence of the inhibitor (final inhibitor concentration of 1 mM). Crystals were grown by micro-seeding at 20°C in 130 nl of sitting drops, mixing 96 nl of protein solution with 10 nl of micro-seed solution (crystals prepared from the same precipitant) and 24 nl of a reservoir solution containing 2.1 M Na-formate (pH 7.0), 0.1 M Bis–Tris (pH 7.0). On mounting, crystals were cryoprotected with reservoir solution mixed with 25% glycerol and vitrified in liquid nitrogen.

For the rebastinib complex, CDK16 protein was buffered in 25 mM HEPES (pH 7.5), 150 mM NaCl, 5% glycerol and 10 mM DTT. CDK16 at 5 mg/ml was mixed with an 1.5 mM inhibitor and concentrated further to 16.4 mg/ml. Crystals were grown at 20°C in 200 nl of sitting drops, mixing 150 nl of protein solution with 50 nl of a reservoir solution containing a 25% PEG smear (PEG2000, PEG3350, PEG4000 and PEG5000MME) and 0.1 M citrate (pH 5.5). Before mounting, crystals were cryoprotected with mother liquor supplemented with an additional 25% ethylene glycol and vitrified in liquid nitrogen.

### Structure determination

Diffraction data for the CDK16 complex with indirubin E804 were collected on one crystal at 100 K on beamline I03 of the Diamond Light Source. Data were integrated with XDS [25] and scaled with SCALA [26]. The structure was solved by molecular replacement with PHASER [27] using the structure of CDK5 (PDB ID: 1UNL) as a search model. Refinement was performed with BUSTER [28] combined with manual rebuilding in COOT [29]. The quality of the model was validated with MOLPROBITY [30].

Diffraction data for the CDK16 complex with rebastinib were collected on one crystal at 100 K on Diamond Light Source beamline I02. Data were indexed and integrated using MOSFLM [31] and then scaled with AIMLESS [32]. Initial phases were calculated in PHASER using the first CDK16 structure as a model for molecular replacement [27]. After obtaining the initial electron density maps by rigid body refinement in REFMAC5 [33], a structural model was generated by automated building in Buccaneer [34]. Several rounds of non-crystallographic symmetry restrained refinement were then performed in REFMAC5 [33] combined with manual modelling with COOT [29]. The refined structures were validated with MolProbity [30]. Structure figures were prepared with PyMOL [35].

## Results

### CDK16 binds type I and type II kinase inhibitors

**Figure 1.**
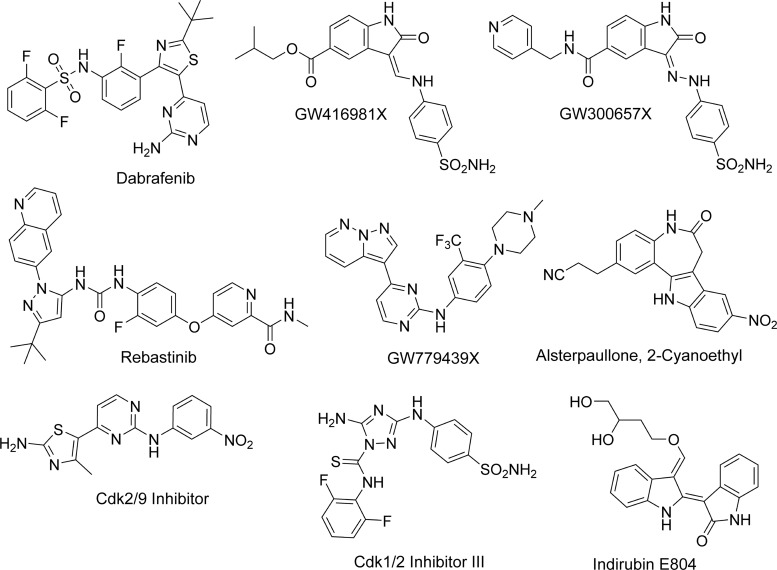
Chemical scaffolds of identified CDK16 inhibitors. Compounds shown induced a *T*_m_ shift for CDK16 of >7°C.

To identify protein constructs suitable for structural studies, human CDK16 was recombinantly expressed in *E. coli* with a variety of N- and C-terminal truncations. A viable construct CDK16^163–478^ was identified that was amenable to crystallisation when expressed with a C-terminal hexahistidine tag and the activation segment mutation S319D. To identify potential inhibitors of CDK16, we screened the recombinant protein kinase domain against many inhibitor libraries using differential scanning fluorimetry (DSF). Ligands in this assay increase a protein's melting temperature (*T*_m_ shift, Δ*T*_m_) by an amount proportional to their binding affinity. A *T*_m_ shift of 8°C or higher is typically observed for compounds binding with a *K*_D_ value of 100 nM or less [36]. Screening against signalling inhibitors obtained from Calbiochem revealed that the most potent hits were known type I inhibitors of the CDK family, confirming the similarity of CDK16 to classical CDKs such as CDK2 (Table 1 and Supplementary Table S1). These included the triazolo-diamine compound Cdk1/2 Inhibitor III (Δ*T*_m_ = 9.6°C), Alsterpaullone, 2-Cyanoethyl (Δ*T*_m_ = 7.9°C), Indirubin E804 (Δ*T*_m_ = 7.6°C) and the aminopyrimidinyl compound Cdk2/9 Inhibitor (Δ*T*_m_ = 7.2°C; Figure 1). A similar preference was observed upon screening of the PKIS (Table 1 and Supplementary Table S2) [37]. Again, the most potent hits with *T*_m_ shift values above 8°C were known CDK2 inhibitors, including the oxindoles GW300657X and GW416981X, as well as the pyrazolo[1,5-*b*]pyridazine compound GW779439X (Figure 1) [37].

**Figure 2.**
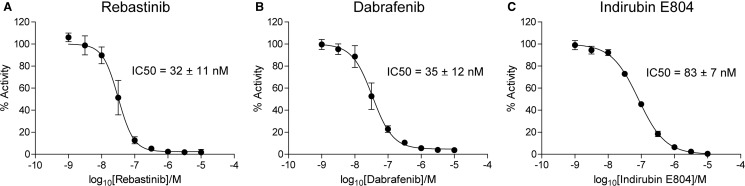
*In vitro* inhibition of CDK16 activity. *E. coli*-purified CDK16 (125 ng) was incubated with rebastinib (**A**), dabrafenib (**B**) or indirubin E804 (**C**) for 5 min followed by the addition of COS1-purified FLAG-cyclin Y (125 ng) and assayed for kinase activity using PCTAIRE-tide as described in ‘Materials and Methods’. Curves were fitted using non-linear regression to the log (inhibitor) vs. response (variable slope) equation using the Graphpad Prism software. Results are expressed as mean ± SD and are shown as percentage activity relative to non-treated kinase, and represent the average of three independent experiments performed using the same protein preparations.

**Figure 3.**
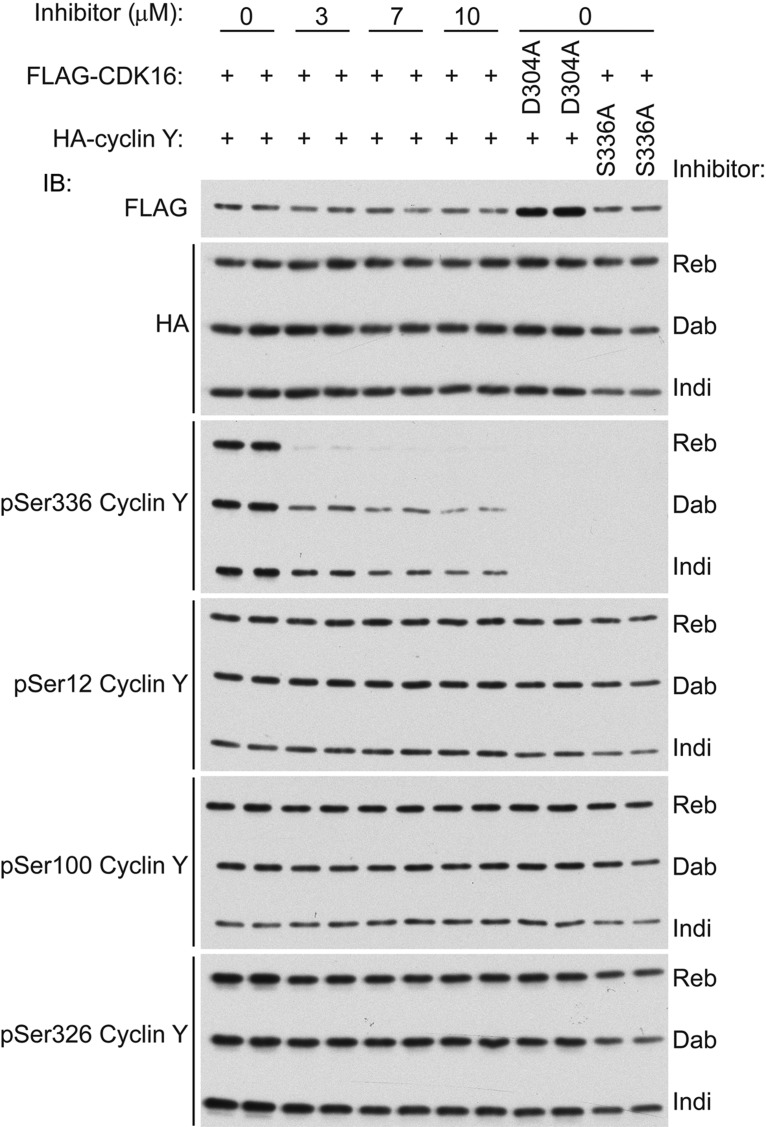
Cellular inhibition of CDK16. HA-cyclin Y WT or S336A mutant was co-transfected with FLAG-CDK16 WT or D304A kinase-inactive mutant in COS1 cells as indicated. Cells were treated for 1 h with varying concentrations of the indicated inhibitors, and cell lysates were immunoblotted using the indicated antibodies. FLAG immunoblot is representative of all three inhibitors. For other immunoblots (HA, pSer12, pSer100, pSer326 and pSer336), three separate blots (Reb, Dab and Indi) were exposed in the same X-ray film following incubation with enhanced chemiluminescence reagent. Reb, rebastinib; Dab, dabrafenib; Indi, indirubin E804.

**Figure 4.**
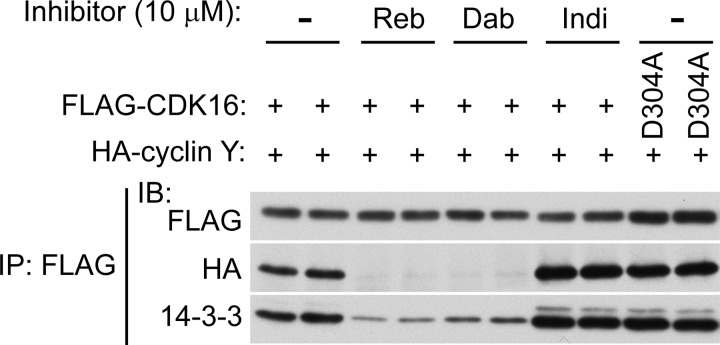
Rebastinib and dabrafenib inhibit CDK16–cyclin Y binding. HA-cyclin Y WT was co-transfected with FLAG-CDK16 WT or D304A kinase-inactive mutant in COS1 cells as indicated and cells were treated for 1 h with 10 μM of the indicated inhibitors (same lysates as in Figure 3). Cell lysates were immunoprecipitated with FLAG-agarose and immunoblotted using the indicated antibodies. Reb, rebastinib; Dab, dabrafenib; Indi, indirubin E804.

**Figure 5.**
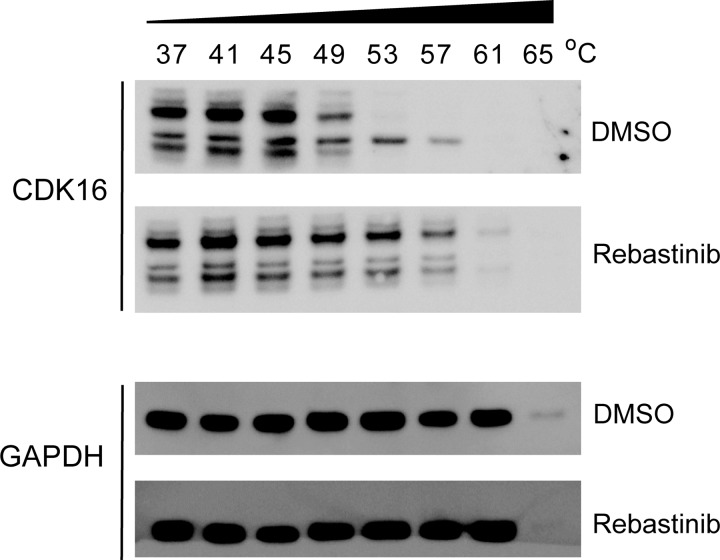
Rebastinib binding to endogenous CDK16 is confirmed by CETSA. Aliquots of IGR-37 melanoma cells were treated with either rebastinib (20 µM) or vehicle (DMSO) and then heated for 3 min at indicated temperatures. CDK16 and GAPDH protein levels were then assessed by IB. Immunoreactive bands specific for CDK16 were confirmed using CDK16 knockdown cells; the middle band of the three observed was found to be non-specific (Supplementary Figure S1).

**Table 1:** Top hits from DSF screening

Calbiochem collection	*T*_m_ shift (°C)	PKIS collection	*T*_m_ shift (°C)	Clinical inhibitors	*T*_m_ shift (°C)
Cdk1/2 Inhibitor III	9.6	GW300657X	8.8	Rebastinib	12.6
Alsterpaullone, 2-Cyanoethyl	7.9	GW416981X	8.7	Dabrafenib	10.4
Indirubin E804	7.6	GW779439X	8.1	SNS-032	7.0
Cdk2/9 Inhibitor	7.2	GW300660X	6.8	Milciclib	6.2
Aurora/Cdk Inhibitor	6.7	GW301784X	6.0	AZ23	5.8
(*Z,E*)-3-(imidazol-4-ylmethylene)indolin-2-one	6.7	GSK238583A	5.8	AT9283	5.7
Indirubin-3′-monoxime-5-sulphonic acid	6.7	SB-732881-H	5.5	Ponatinib	5.5
Flt3 Inhib III	6.6	SB-739245-AC	5.4	Dinaciclib	4.8
5-Iodo-indirubin-3′-monoxime	6.1	SB-732881	5.2	PIK-75	4.7
SU9516	6.0	GW780056X	4.8	Regorafenib	4.1

We also screened ∼150 kinase inhibitors (FIMM drug collection) that have either entered clinical trials or been approved by the US Food and Drug Administration. While many moderate binders were observed, two compounds produced exceptional *T*_m_ shift values far exceeding those from the previous screening (Table 1 and Supplementary Table S3). The most potent hit was rebastinib (DCC-2036), which yielded a *T*_m_ shift of 12.6°C. Rebastinib is a multitargeted type II kinase inhibitor that was developed to inhibit BCR-ABL as well as the drug-resistant gatekeeper mutant ABL^T315I^ (Figure 1) [25]. It has entered phase I clinical trials for chronic myeloid leukaemia [25]. The other significant hit was dabrafenib (GSK2118436), which showed a *T*_m_ shift of 10.4°C. Dabrafenib is an ATP-competitive type I inhibitor of mutant BRAF^V600E^ that has been approved for clinical use in advanced melanoma [26]. Other clinically relevant inhibitors yielding a *T*_m_ shift of 5°C or more included many CDK inhibitors, such as SNS-032 (7.0°C), milciclib (6.2°C) and dinaciclib (5.5°C). Thus, CDK16 has a propensity to bind both type I and type II inhibitors and has a broad preference for inhibitors targeted to other CDK kinases (Figure 1).

### Rebastinib and dabrafenib are potent inhibitors of CDK16

Shehata *et al*. [5] demonstrated previously that recombinant FL CDK16 from bacterial expression can be activated by COS1-purified recombinant cyclin Y, which co-purifies with endogenous 14-3-3. We therefore used this activation strategy to perform an *in vitro* kinase assay to determine the IC_50_ values of selected inhibitors. Varying concentrations of each compound were preincubated with *E. coli*-purified CDK16 for 5 min, followed by the addition of COS1-purified cyclin Y/14-3-3 complex. CDK16 was then assayed for phosphotransferase activity against the optimised substrate PCTAIRE-tide (PKSPKARKKL) (Figure 2) [22]. Rebastinib and dabrafenib showed comparable IC_50_ values of 32 and 35 nM, respectively, whereas indirubin E804 exhibited weaker inhibition with an IC_50_ value of 83 nM. Overall, these data are in good agreement with the DSF assay results, which yielded the same rank order of potencies (Table 1).

### Rebastinib inhibits CDK16 activity in cells

We next tested the ability of the compounds to inhibit CDK16 activity in intact cells. It was shown recently that Ser336 phosphorylation on cyclin Y is dependent on CDK16 kinase activity [5]. We therefore tested whether inhibitor treatment would affect Ser336 phosphorylation using a phospho-specific antibody. HA-tagged cyclin Y WT or S336A mutant was co-expressed with FLAG-tagged CDK16 WT or D304A (kinase-inactive) mutant in COS1 cells. The cells were treated with increasing concentrations of the respective inhibitors (rebastinib, dabrafenib or indirubin E804) for 1 h, and lysates were immunoblotted for detection of Ser336–cyclin Y phosphorylation. As shown in Figure 3, Ser336 phosphorylation decreased in a dose-dependent manner with all of the inhibitors tested. While rebastinib and dabrafenib showed comparable IC_50_ values in cell-free assays, rebastinib treatment resulted in a significantly more potent inhibitory effect in COS1 cells compared with dabrafenib and indirubin E804. No detectable Ser336 phosphorylation was observed in the kinase-inactive (D304A) CDK16 or phospho-deficient (S336A) cyclin Y mutant controls, confirming the requirement for CDK16 activity and specificity of the phospho-Ser336–cyclin Y antibody as recently reported [5]. To further demonstrate the specificity of the inhibitors, we tested the phosphorylation status of three other sites within the cyclin Y protein that are known to be independent of CDK16 activity [5]. Importantly, there was no significant change in the phosphorylation of Ser12, Ser100 or Ser326, confirming that the inhibitors were acting specifically through CDK16 (Figure 3).

The three tested inhibitors are expected to have three different binding modes that may differentially affect the binding of CDK16 to the cyclin Y/14-3-3 complex. While indirubin E804 is compatible with an active kinase conformation [38], rebastinib and dabrafenib are expected to bind to inactive ‘DFG-out, αC-in’ and ‘DFG-in, αC-out’ kinase conformations, respectively, that may hinder cyclin Y association [39,40]. To test this hypothesis, we immunoprecipitated FLAG-tagged CDK16 from variously treated COS-1 cells and performed IB for HA-tagged cyclin Y and endogenous 14-3-3 protein. Both CDK16 WT and the D304A kinase-inactive mutant were able to immunoprecipitate cyclin Y and 14-3-3 protein, confirming that their association with CDK16 was independent of CDK16 kinase activity (Figure 4). However, the amounts of cyclin Y and 14-3-3 in these immunoprecipitates were dramatically reduced upon prior treatment of the cells with either rebastinib or dabrafenib (Figure 4). The low amounts of 14-3-3 protein still detected in these immunoprecipitates potentially indicate some direct association of the 14-3-3 with CDK16 [7]. In contrast, treatment with indirubin E804 had no effect (Figure 4). These data strongly suggest that rebastinib and dabrafenib induce structural changes in the monomeric CDK16 that sterically interferes with its binding to cyclin Y.

The binding of rebastinib to endogenous CDK16 was additionally confirmed by a CETSA, a recently developed methodology which takes advantage of alterations in protein thermal stability upon ligand binding [23,24]. Aliquots of IGR-37 melanoma cells were treated with either rebastinib (20 µM) or vehicle (DMSO) and then heated for 3 min at different temperatures ranging from 37 to 73°C. CDK16 protein levels were then assessed by IB. In the DMSO-treated control cells, CDK16 was stable up to 49°C, whereas rebastinib protected CDK16 from unfolding at higher temperatures up to 57°C (Figure 5). We verified that the immunoreactive bands (CDK16) were specific using CDK16 knockdown cells as controls (Supplementary Figure S1). These results are consistent with the large thermal shift observed in the initial compound library screen (Table 1).

### Structure determination

Crystallisation trials using the CDK16 kinase domain were performed with a number of the inhibitors identified from the initial compound screening. Viable crystals were first obtained for a complex containing the type I inhibitor indirubin E804. The structure of this complex was solved using CDK5 as a phasing model and refined at a resolution of 2.4 Å with one CDK16 monomer in the asymmetric unit. The electron density maps for the structure were generally of high quality. The CDK16 chain was modelled between residues Met162–Glu473, with the exception of regions of poor electron density at the αC helix, Glu199–Ala204, as well as the activation segment Ser312–Val323. The C-terminus between Ala474–His485, including the affinity tag, could also not be built. There was clear density for the indirubin E804 inhibitor in the ATP-binding pocket.

We subsequently obtained crystals of CDK16 with the type II inhibitor rebastinib and were able to solve the structure of this complex at a resolution of 2.2 Å (see Table 2 for the data collection and refinement statistics for both structures). The electron density maps were similarly of high quality and showed two CDK16 monomers in the asymmetric unit. CDK16 chain A was modelled between residues Met162–Ala474, with the exception of regions of poor electron density in the activation segment Ser312–Lys316. CDK16 chain B was modelled between residues Met162–Ser475, except for residues Lys311–Val322. Both protein chains showed clear electron density in the ATP-binding pocket for rebastinib. Viable crystals were not obtained with dabrafenib.

**Table 2:** Diffraction data collection and refinement statistics (molecular replacement) Values in parentheses are for highest resolution shell. Abbreviation: R.m.s, root-mean-square.

	CDK16–indirubin E804 (PDB ID: 3MTL)	CDK16–rebastinib (PDB ID: 5G6V)
Data collection		
Beamline	Diamond light source, I03	Diamond light source, I02
Wavelength (Å)	0.9800	0.9174
Space group	*P*4_3_2_1_2	*P*2_1_22_1_
Cell dimensions		
*a*, *b*, *c* (Å)	47.4, 47.4, 341.3	57.2, 86.9, 146.3
*α*, *β*, *γ* (°)	90.0, 90.0, 90.0	90.0, 90.0, 90.0
Number of unique reflections	16 459 (2304)	37 175 (3157)
Resolution (Å)*	20.09–2.40 (2.53–2.40)	28.98–2.20 (2.27–2.20)
*R*_merge_	0.102 (0.684)	0.093 (0.454)
CC(1/2)	0.996 (0.909)	0.996 (0.818)
*I*/σ*I*	9.6 (2.1)	9.4 (2.8)
Completeness (%)	99.8 (100)	98.3 (97.4)
Redundancy	6.7 (6.8)	5.2 (4.9)
Refinement		
Ligand	Indirubin E804	Rebastinib
Resolution (Å)	20.09–2.40 (2.57–2.40)	86.94–2.20 (2.26–2.20)
Number of reflections	16 358	35 328
*R*_work_/*R*_free_	19.4/26.3	20.8/27.1
Average *B* factor (Å^2^)	72.5	40.433
R.m.s. deviations		
Bond lengths (Å)	0.010	0.009
Bond angles (°)	1.21	1.34
Molprobity		
Ramachandran favoured (%)	95.5	96.0
Ramachandran allowed (%)	4.5	4.0

* Single crystal.

### The isolated CDK16 kinase domain shows conformational plasticity

**Figure 6.**
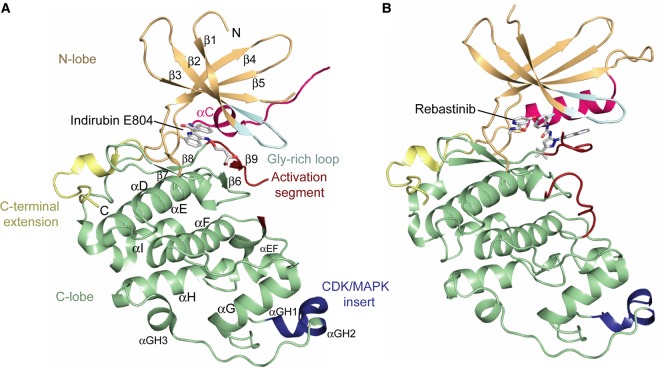
Structures of the CDK16 kinase domain. Structure of the CDK16 kinase domain solved in complex with indirubin E804 (**A**) or rebastinib (**B**). Selected CDK16 motifs are labelled and highlighted by different colours.

The crystal structures of monomeric CDK16 represent the first structures from the PCTAIRE family of CDK kinases (Figure 6). The kinase domain exhibits the classical bilobal architecture intermixed with short insertions that help to define the CDK family fold. The complex with indirubin E804 displays some disorder in the kinase N-lobe, consistent with its requirement for a cyclin partner. This lobe features a five-stranded β-sheet and the αC helix, which is expected to contain the diverged ‘PCTAIRE’ cyclin-binding sequence motif. However, in the indirubin E804 complex, the αC is reduced to a single helical turn and the preceding PCTAIRE motif instead adopts a loosely extended conformation that is stabilised by crystal packing. In contrast, the rebastinib complex has a canonical αC helix that includes the PCTAIRE motif, which probably reflects the additional interactions this region forms with the bound inhibitor. Indeed, the complete N-lobe is well defined in this structure, including the flexible β3–αC loop insertion that typically supports the interaction of a CDK with its cyclin. This surface is also stabilised by crystal packing interactions. The predominantly α-helical C-lobe contains the CDK/MAPK insert, which packs against the αDE and αG helices, as well as a PCTAIRE family specific C-terminal extension that stretches behind the αI helix before folding back to terminate beside the αD helix (Figure 6).

**Figure 7.**
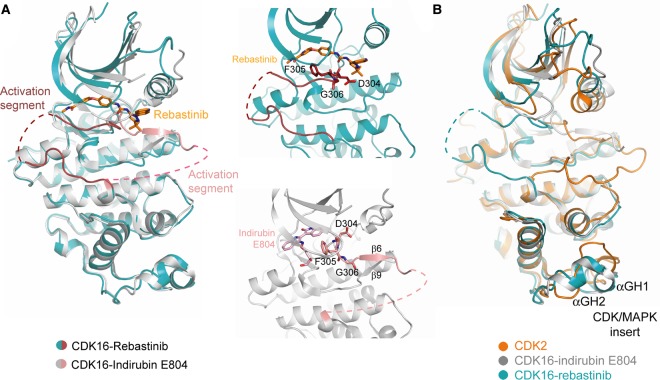
CDK16 structures show different conformations. (**A**) Superposition of the different CDK16 structures (left panel) shows the alternative conformations adopted by the activation segment when in complex with indirubin E804 (type I inhibitor) or rebastinib (type II inhibitor). Right panel shows the respective positions of residues in the ‘DFG motif’. (**B**) Superposition of CDK2 (PDB ID: 5IF1 [59]) and CDK16 structures reveals structural differences in the CDK/MAPK insert. Colours are indicated by the keys provided.

Comparison of the two complexes reveals small differences in the conformations of the N-lobes and marked differences in the folding of the activation segments (Figure 7A). The rebastinib co-structure displays an inactive ‘DFG-out, αC-in’ configuration. In this structure, Asp304, Phe305 and Gly306 are flipped into an inverted conformation relative to an active kinase. The activation segment therefore folds antiparallel to the β1 strand and occludes the front of the ATP pocket. In contrast, in the indirubin E804 complex, this segment extends below the αC helix to form the expected β6–β9 sheet (Figure 7A). However, a partial inversion of the DFG motif is still observed in which Asp304 and Phe305 are flipped out, while Gly306 retains its expected conformation. Thus, this structure does not conform to a classic type I inhibitor complex. This atypical feature further suggests that CDK16 is only loosely structured in the absence of cyclin Y and ordered by the bound inhibitor. For example, in both complexes, the inhibitors help to stabilise the critical salt bridge between the catalytic lysine (Lys194, β3) and Glu211 (αC). Despite this interaction, the inactive conformations shown by both kinase structures would not support substrate recruitment.

As well as the variant PCTAIRE motif, the CDK16 fold shows differences to other CDKs in the conformation of the CDK/MAPK insert (Figure 7B). The CDK/MAPK insert forms an additional protein–protein interaction surface in the CDK family. The insert in CDK16 contains a well-defined short helix αGH_1_, whereas the CDK2 insert is predominantly a loop (defined as ‘L14’) best known for its interaction with CKS1 (Figure 7B) [41]. Thus, the surfaces of CDK2 and CDK16 probably evolved to mediate distinct protein–protein interactions.

### Structural basis for inhibitor-binding interactions

**Figure 8.**
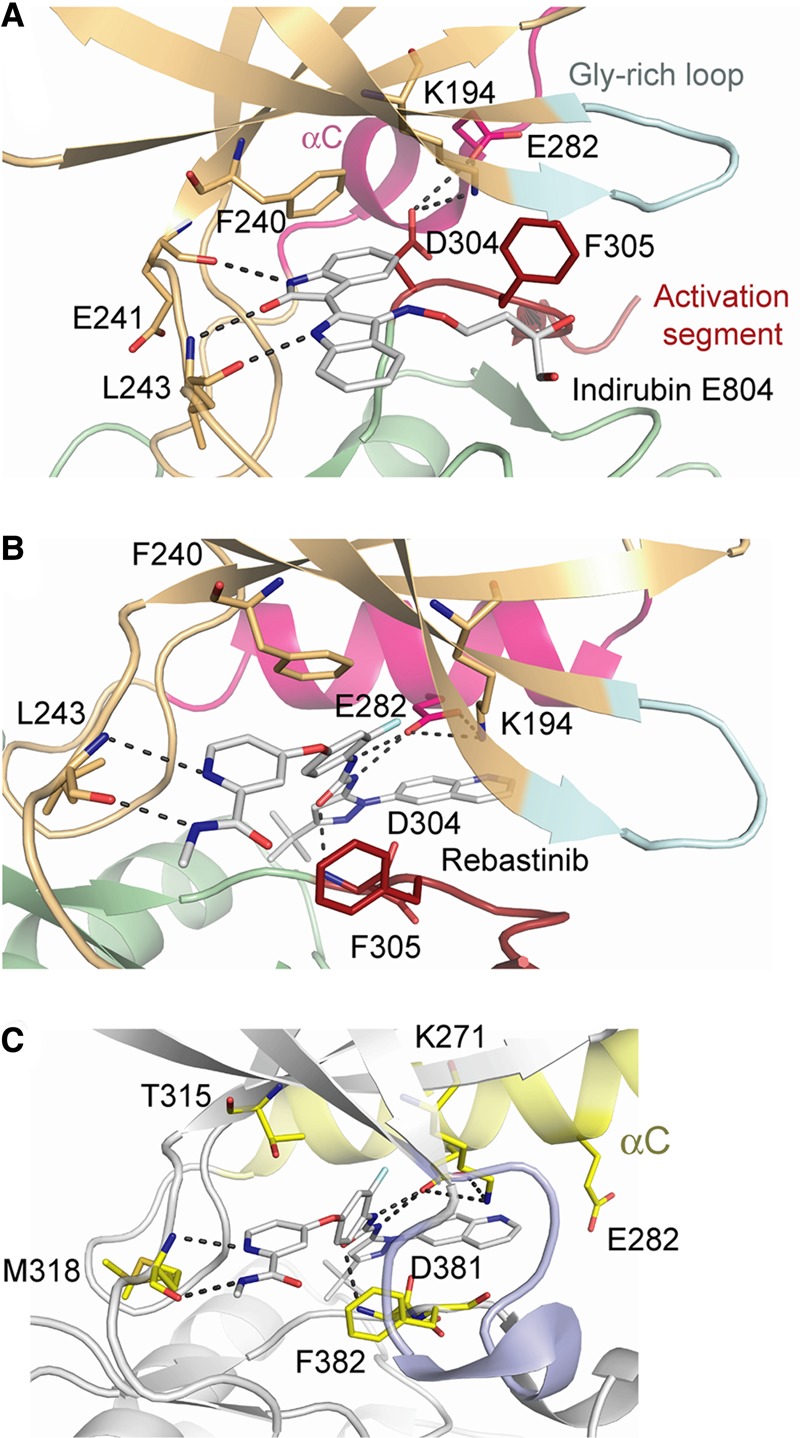
Interactions of indirubin E804 and rebastinib with the ATP-binding pocket of CDK16. (**A**) Indirubin E804 binds to the kinase hinge region of CDK16 forming three hydrogen bonds. An atypical induced fit is observed in which the activation segment residue Phe305 is flipped out to establish a cage-like structure around the inhibitor. (**B**) Rebastinib binds as a type II inhibitor forming a total of five hydrogen bonds with the ATP-binding pocket of CDK16. The binding is facilitated by an inverted conformation of the ‘DFG motif’, which creates an extra pocket for inhibitor binding below the αC helix. (**C**) Structure of the ABL complex with rebastinib for comparison (PDB ID: 3QRI [39]). The glycine-rich loop shows a collapsed conformation that forms a cage around the inhibitor. Selected structural motifs are labelled and highlighted by different colours.

The indirubin E804 scaffold is largely planar and binds to the kinase hinge region of CDK16 using three hydrogen bonds, including two from the indole-2-one moiety and one from the indole moiety (Figure 8A). These groups also present a significant hydrophobic surface for van der Waals interactions with the ATP pocket residues Leu171, Val179, Ala192, Val224, Phe240, Leu293 and Ala303. The hydroxybutyloxime moiety extends across the expected ribose- and phosphate-binding sites of the ATP pocket. Here, the flipped-out conformation of Phe305 in the activation segment establishes a cage-like structure around the inhibitor. The binding therefore features an induced fit that maximises the kinase–inhibitor interaction.

The extended binding mode of the type II inhibitor rebastinib is quite distinct from that of the type I inhibitor, indirubin E804 (Figure 8B). As anticipated, the binding of rebastinib is facilitated by an inverted conformation of the ‘DFG motif’, which creates an extra pocket for inhibitor binding below the αC helix. Rebastinib forms a total of five hydrogen bonds with the ATP-binding pocket of CDK16, as similarly observed in the equivalent ABL structure (Figure 8C) [39]. The carboxamide-substituted pyridine ring forms two hydrogen bonds with the backbone amide and carbonyl of the ATP-hinge residue, Leu243. The urea moiety's nitrogens also form two hydrogen bonds with Glu211 from the αC ‘PCTAIRE’ motif. A further hydrogen bond is formed between the backbone amide of Asp304 and the urea carbonyl (Figure 8B). The central phenyl ring is sandwiched between the gatekeeper residue Phe240 and the flipped-out Phe305 (DFG motif), providing strong π–π packing interactions. The *t*-butyl group occupies the hydrophobic pocket vacated by the inverted Phe305. Finally, the quinoline moiety extends below Glu211 and the αC helix (Figure 8B). CDK16 and ABL show many side chain differences in this region that could be useful for the future modification of rebastinib to more selectively target CDK16. For example, the binding of the quinoline moiety in ABL is capped by Glu282 at the N-terminus of the αC (Figure 8C), whereas the smaller Thr207 side chain in CDK16 would allow the quinoline to be expanded.

## Discussion

For many years, the position of CDK16 (PCTAIRE1) within the CDK family was uncertain: the identity of its cyclin partner remained elusive and its existence was cast in doubt [7]. Now, cyclin Y has been implicated in binding to both the PCTAIRE1–3 (CDK16–18) and related PFTAIRE1–2 (CDK14–15) family kinases [3,6,42]. Taken together, these kinases belong with CDK5 to an atypical group of CDK proteins related to the yeast kinase Pho85 [2]. Unusually, these CDKs bind to cyclin partners that are predicted to contain only a single cyclin box domain, whereas most cyclins have two domains. The crystal structures of CDK16 reported here offer the first structural view of a kinase from either of the PCTAIRE or PFTAIRE subfamilies (CDK14–18). Overall, the scaffold of CDK16 bears all of the structural elements expected of a CDK. Furthermore, its cyclin dependency is strongly supported by the inactive and partly disordered conformation found in the indirubin E804 complex. The localised unfolding of the PCTAIRE motif in the αC helix is intriguing and quite unlike the structures of other monomeric CDKs. Other unusual features in the CDK16 structure include a partially inverted DFG motif and distinct conformations of the large C-terminal extension and the CDK/MAPK insert. Taken together, these features may suggest an additional stabilising role for the uncharacterised N-terminal extension of CDK16 or perhaps a missing 14-3-3 protein interaction, which appears necessary for the activation of both the PCTAIRE and PFTAIRE family kinases [5,8].

The conformational plasticity shown by CDK16 supports its binding to the diverse set of chemical scaffolds identified here, including both type I and type II inhibitors. The observed enrichment of known CDK inhibitors is consistent with both the sequence conservation, and the large amount of chemistry that has been directed towards this family. None of the identified scaffolds are reported to be highly selective, suggesting that additional chemistry would be required to develop a compound for use as a probe of CDK16 function in cells. Most interestingly, we identified two clinically tested inhibitors, dabrafenib and rebastinib, as the most potent CDK16 inhibitors both *in vitro* and in intact cells. Thus, there is potential benefit to test these compounds in the future in cancers associated with CDK16 overexpression [16]. However, given that dabrafenib and rebastinib are not specific inhibitors of CDK16, the identification or development of CDK16-selective inhibitors may be necessary.

We also found that the binding of CDK16 to dabrafenib and rebastinib was mutually exclusive with its binding to the cyclin Y/14-3-3 complex. Such a mechanism has been previously reported for inhibitors of the CDK2 kinase. For example, Deng *et al*. [43] have identified a quinoline-based inhibitor that induces an αC-out conformation in CDK2 and therefore disrupts the proper interaction of the PSTAIRE motif with cyclin A. Alexander *et al*. [44] have additionally identified many type II inhibitors that bind to a previously unrecognised DFG-out conformation of CDK2 and similarly block its association with cyclin A by removing the potential for an interaction with the CDK2 activation segment. Dabrafenib and rebastinib are expected to disrupt the CDK16 interaction with the cyclin Y/14-3-3 complex via similar structural mechanisms. Dabrafenib is expected to induce an αC-out kinase conformation, as observed in various BRAF complexes [40,45], whereas the co-structure of CDK16 and rebastinib has confirmed a DFG-out binding mode for this type II inhibitor. Thus, structural changes in either of the N- or C-terminal lobes of CDK16 appear to affect the binding of cyclin Y/14-3-3. This is consistent with the homologous structures of CDK5 [46] and Pho85 [47], which have revealed extensive cyclin interactions across both kinase lobes, as also observed in the CDK2/cyclin A complex [48].

The assembly of the active CDK16/cyclin Y/14-3-3 complex is tightly regulated by phosphorylation sites within the FL proteins that remain to be structurally characterised. Once activated, the CDK16 kinase performs critical functions within the brain and testis, including neurite outgrowth [7], retrograde axonal trafficking [10], exocytosis [11], vesicle transport [12] and spermatogenesis [3]. Furthermore, CDK14, CDK16, CDK18 and cyclin Y have all been linked to cancer cell proliferation, migration or cancer drug resistance [18,49–54]. Moreover, these effects were reversed by siRNA-mediated knockdown of the respective target [19,20,54–58]. The excellent druggability of CDK16 and chemical scaffolds identified here provide further opportunity to better characterise the role of these kinases and suggest the potential to develop more selective CDK16 inhibitors for possible clinical use and as powerful basic research tools.

## Database deposition

The atomic co-ordinates and structure factors (codes 3MTL and 5G6V) have been deposited in the Protein Data Bank (http://www.rcsb.org).

## Abbreviations

CDK16: cyclin-dependent kinase 16
CETSA: cellular thermal shift assay
CKS1: cyclin-dependent kinases regulatory subunit 1
DMSO: dimethyl sulfoxide
DSF: differential scanning fluorimetry
DTT: dithiothreitol
FBS: foetal bovine serum
FL: full length
GAPDH: glyceraldehyde-3-phosphate dehydrogenase
HA: haemagglutinin
IB: immunoblotting
MAPK: mitogen-activated protein kinase
PBS: phosphate-buffered saline
PKIS: published kinase inhibitor set
PMSF: phenylmethylsulphonyl fluoride
TCEP: tris(2-carboxyethyl)phosphine
TLCK: Tosyl-L-lysyl-chloromethane hydrochloride
WT: wild type.
